# Aberrant DNA Methylation in Esophageal Squamous Cell Carcinoma and its Clinical Implications in Systemic Chemotherapy

**DOI:** 10.7150/ijms.109161

**Published:** 2025-02-03

**Authors:** Zhao Li, Xi Chen, Yongkang Li, Yan Xu, Yang Zhou

**Affiliations:** 1 Department of Health Prevention and Care, Beijing Hospital, National Center of Gerontology, Institute of Geriatric Medicine, Chinese Academy of Medical Sciences, No. 1 Dahua Road, Beijing 100730, China.; 2 Department of Oncology, Beijing Hospital, National Center of Gerontology, Institute of Geriatric Medicine, Chinese Academy of Medical Sciences, No. 1 Dahua Road, Beijing, 100730, China.; 3 Graduate School of Peking Union Medical College, Chinese Academy of Medical Sciences, No. 5 Dong Dan San Tiao, Beijing, 100005, China.; 4 Department of Thoracic Surgery, National Cancer Center/National Clinical Research Center for Cancer/Cancer Hospital, Chinese Academy of Medical Sciences and Peking Union Medical College, Panjiayuan Nanli No.17, Beijing 100021, China.

**Keywords:** esophageal squamous cell carcinoma, DNA methylation, systemic chemotherapy, therapeutic response, epigenetic therapy

## Abstract

Esophageal cancer is a significant global health concern, with esophageal squamous cell carcinoma being the predominant subtype in high-incidence regions like China. Despite advances in multidisciplinary treatments, the prognosis for ESCC remains poor, with systemic chemotherapy facing the challenge of drug resistance. Epigenetic alterations, particularly DNA methylation, play a crucial role in ESCC carcinogenesis and therapeutic response. Aberrant DNA methylations, including global hypomethylation and promoter-specific hyper-methylation, disrupt critical pathways such as cell cycle regulation, apoptosis, and DNA repair, contributing to chemoresistance. Several studies have identified methylation markers that predict treatment response, particularly for chemotherapy, targeted therapy and immunotherapy, such as *p16* and *GPX3* for cisplatin, *MTHFR* for 5-FU, *CHFR* for paclitaxel. DNA methyltransferase inhibitors and other epigenetic therapies are being explored to reverse these methylation changes and enhance therapeutic efficacy. However, the clinical utility of these markers remains limited due to the lack of large-scale validation and concerns over off-target effects. This review aims to summarize all aberrant methylation alterations in ESCC and the clinical implications of aberrantly methylated candidate genes identified in ESCC systemic chemotherapy, with the goal of further understanding the underlying molecular mechanisms, refining methylation-targeting therapies, and integrating them with conventional treatments to improve patient outcomes.

## Introduction

Esophageal cancer ranks as the 11th most common newly diagnosed cancer and is the 7th leading cause of cancer mortality worldwide, with approximately 511,000 new cases and 445,000 deaths reported in 2022 according to the latest global cancer statistics 1. Esophageal cancer (EC) is histologically classified into esophageal squamous cell carcinoma (ESCC), esophageal adenocarcinoma (EAC) and other subtypes 2. ESCC and EAC have significant differences in many features such as geographic distribution, etiologies, pathogenesis, physiological and molecular mechanisms 3-6. Cigarette smoking, alcoholic abuse and inappropriate dietary pattern (e.g. nutritional deficiencies, nitrosamines, betel quid chewing, pickled vegetables and hot food/beverages) are the major risk factors for ESCC 5, 7. Meanwhile EAC accounts for nearly two-thirds of esophageal cancer cases in high-income countries with excess body weight, gastroesophageal reflux disease, and Barrett's esophagus as the major risk factors 7. Esophageal squamous cell carcinoma is the predominant subtype in China, accounting for approximately 90 percent of esophageal cancer and ranking the 7th in incidence and 5th in mortality 8. Multidisciplinary treatments including surgery, radiotherapy, chemotherapy, molecular-targeting therapy and immunotherapy have improved overall survival of esophageal cancer patients, nonetheless the prognosis is still poor with overall 5-year survival ranging from 15% to 25% 9, 10.

Systemic chemotherapy which mainly consists of cytotoxic agents, molecular-targeted agents and immune checkpoint inhibitors play an essential role in the treatment of ESCC patients. Cytotoxic agents, such as cisplatin (DDP) and 5-fluorouracil (5-FU), have been used as the standard treatment for decades 11. However, their limited efficacy due to resistance and inevitable adverse effects significantly impact the long-term survival of ESCC patients. In terms of immunotherapy, the Food and Drug Administration (FDA) has approved the PD-1 inhibitor pembrolizumab for second-line treatment of PD-L1 positive, advanced or metastatic ESCC 12, 13. Based on phase 2 and phase 3 clinical trials, nivolumab as another PD-1 inhibitor was found to outperform conventional chemotherapy irrespective of PD-L1 status 14, 15. Multiple molecular-targeted agents against EGFR, HER-2, VEGFR, c-Met and other oncogenes were also investigated in ESCC treatment. As vital candidates, anti-EGFR inhibitors such as cetuximab and nimotuzumab have some promising results in clinical trials 16, 17. However, most molecular-targeted agents haven't demonstrated clinical utility in randomized phase 3 trials of ESCC so far.

Negative results from multiple systemic chemotherapy clinical trials suggest the importance of identifying predictive biomarkers of responses to specific agents. Nevertheless, definitive predictive biomarkers to determine right patient candidates remain unclear for conventional chemotherapy, targeted therapy and immunotherapy in patients with ESCC. Recently intriguing findings have emerged that both genetic and epigenetic alterations equally contribute to ESCC with collusion between them, which provides novel theories and understandings about therapeutic effect evaluation, potential therapeutic targets and prognostic prediction in ESCC patients 18.

DNA methylation, along with other epigenetic alterations such as histone modification, chromatin remodeling and the expression of microRNAs, plays an important role in transcriptional regulation, thus interfering driver genes expression 19, 20. The best-understood epigenetic mechanism is DNA methylation, which mainly involves global hypomethylation and specific gene promoter hypermethylation especially of tumor suppressor genes (TSGs). Sufficient evidence has suggested that TSGs transcriptional silence by promoter hypermethylation and increased chromosome instability by global hypomethylation were critical steps in ESCC carcinogenesis 21. Besides, epigenetic alterations of upstream transcription factors have been proved to cause tumor suppressor network silence as well. Silenced genes with aberrant methylation occurred in almost every significant molecular pathway associated with systemic chemotherapy, such as cell cycle regulation, cell apoptotic, DNA damage repair, cell adhesion and immune response regulation pathways 22.

DNA methylation has a crucial role in esophageal carcinogenesis, which was indicated in previous researches. More specifically, aberrant methylated genes have been detected in fields of early screening, diagnosis, prognosis and chemo-sensitivity prediction. Promising predictive markers have great potentiality to improve limited therapeutic effect and poor prognosis in ESCC. In this review, we aim to summarize the molecular mechanisms of aberrant methylated candidate genes in ESCC systemic chemotherapy and their clinical applications on biomarkers of chemotherapy response and therapeutic potentials.

## Aberrantly methylated genes in ESCC

Numerous DNA methylation profiling researches have revealed that, similar to other cancers, ESCC genome exhibits widespread regions of hypomethylation and focal regions of hypermethylation, when compared with adjacent normal tissues or noncancer controls 23, 24. Although the general patterns of hyper- and hypomethylation are similar, the distribution of specific methylation sites differs from that observed in other related cancers (e.g. head and neck squamous cell carcinoma). Large-scale genomic and epigenomic data further confirm that ESCC and EAC exhibit nearly mutually exclusive sets of driver genes and significantly distinct epigenetic alterations. Hence, a focused summary of aberrant methylation alterations in ESCC is both crucial and indispensable.

**Figure 1:**
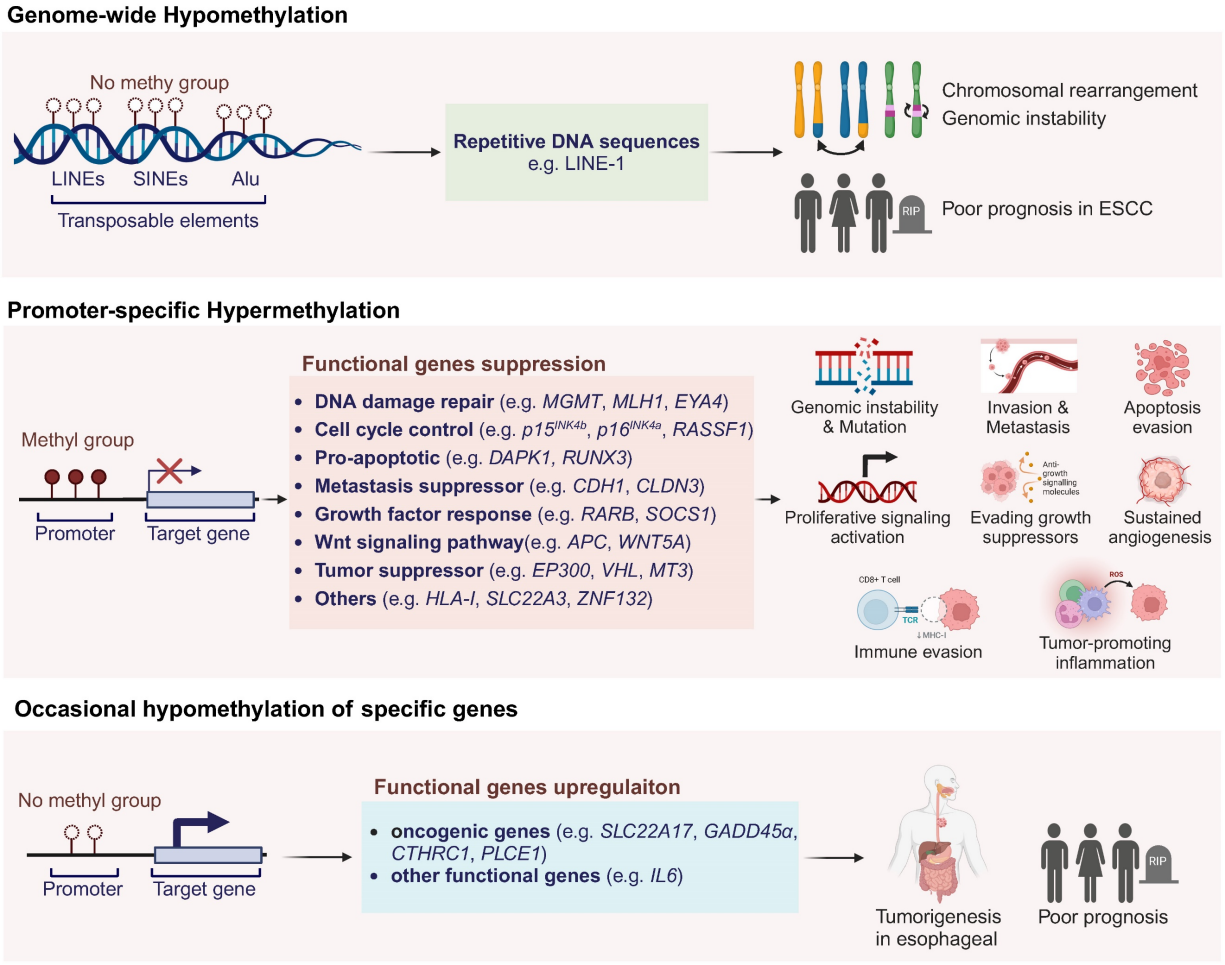
Aberrant DNA methylation patterns in ESCC. Promoter hypermethylation silencing tumor suppressor genes and hypomethylation of repetitive DNA sequences lead to genomic instability and tumorigenesis. Occasional hypomethylation of specific genes were also reported to contribute to ESCC development. Abbreviations: LINEs, long interspersed nuclear elements; SINEs, short interspersed nuclear elements; LINE-1, long interspersed nuclear element-1; ESCC, esophageal squamous cell carcinoma; TCR, T-cell receptor; MHC-I, major histocompatibility complex class I; ROS, reactive oxygen species. Created in BioRender. Li, YK. (2025) https://BioRender.com/z74h803 (accessed on 25 January 2025).

As illustrated in **Figure 1**, these alterations in the epigenetic landscape, characterized by global genomic DNA hypomethylation and specific genes promoter hypermethylation, both contribute to the pathogenesis and development of ESCC via distinct mechanisms 25. Besides those, the occasional hypomethylation of specific genes has also seen some research progress, which will be detailed in the following sections.

### Global genomic DNA hypomethylation

Feinberg et.al 26 reported global genomic DNA hypomethylation in tumor tissues as early as 1983. Gaudet et.al 27 managed to induce tumorigenesis in mice by genomic hypomethylation, which validated the correlation between genomic hypomethylation and tumorigenesis. However, the underlying mechanism of global genomic DNA hypomethylation in ESCC is still equivocal. Current studies showed that DNA hypomethylations, especially those located in long interspersed nuclear elements (LINEs), short interspersed nuclear elements (SINEs) and other highly repetitive DNA sequences like Alu elements, could destabilize chromosome and cause rearrangements 28, 29. LINE-1 elements constitute nearly 17% of human genome as important parts, so methylation of LINE-1 indicates the global DNA methylation level 21. Variable studies showed that LINE-1 methylation in ESCC ranged from 25% to 92%, and low methylation level of LINE-1 was indicated to correlated significantly with poor prognosis 30, 31. Global genomic DNA hypomethylation could still influence tumorigenesis through some other mechanisms that require further investigations 25.

### Promoter-specific hypermethylation in ESCC

DNA methylation has become the best-studied epigenetic field due to the stable characteristic and credible detection techniques. Previously published studies have shown that as much as hundreds of genes have been found to be silenced by promoter hypermethylation. Numbers of evidences have suggested that the promoter hypermethylation of tumor suppressor genes is the critical step in carcinogenesis of ESCC 32. In the Table 1, we summarized all reported TSGs in ESCC which play significant roles in signal pathways including cell-cycle regulation, apoptosis control, cell adhesion, metastasis, DNA damage repair, growth factor response, etc. The other functional genes which are silenced by promoter hypermethylation, accompanied with detailed gene information and corresponding methylation frequency in tumor tissues, were also listed as below.

### Rare hypomethylation of specific genes in ESCC

According to the comprehensive analysis of the DNA methylation profile in ESCC, only 12.4% out of 26081 differentially methylated regions exhibited hypomethylation while 87.6% showed hypermethylation 106. In ESCC carcinogenesis, the phenomenon of occasional hypomethylation of specific genes has been a subject of intense research 38, 106. Cui *et al.* found that the hypomethylation of *NGALR* (*SLC22A17*) gene contributes to its overexpression in ESCC, implicating its role in tumorigenesis 107. *GADD45α*, involved in DNA repair, is also overexpressed due to promoter hypomethylation in ESCC 108. The DNA polymerase iota (*Polι*) gene, overexpressed in ESCC, is linked to hypomethylation, potentially driving genomic instability 109. The *IGF2* gene is influenced by hypomethylation at its DMR0, correlating with poor prognosis 110. Similarly, the *OCT1* and *CTHRC1* genes are upregulated in ESCC due to hypomethylation, activating MAPK/MEK/ERK pathway and facilitating tumor progression 111, 112. The hypomethylation-associated upregulation of *PLCE1* has been linked to tumorigenesis, tumor angiogenesis and poor prognosis in ESCC 113. The *IGFBP7* gene shows lower promoter methylation, with its unmethylated state correlating with increased globulin levels and reflux in ESCC patients 114. Notably, the gene *CDKN1C*, a putative tumor suppressor, exhibits diminished expression associated with the loss of methylation at the differentially methylated region (DMR)-LIT1, rather than its own promoter CpG methylation 115. Lastly, the interleukin 6 (*IL6*) gene, which is known to activate the JAK/STAT3 pathway, is implicated in ESCC and influenced by hypomethylation 45. These findings underscore the complex interplay between hypomethylation and the dysregulation of key genes in ESCC pathogenesis.

## Clinical implications of aberrantly methylated genes in systemic chemotherapy

### DNA methylation and therapeutic response prediction in ESCC

**Figure 2:**
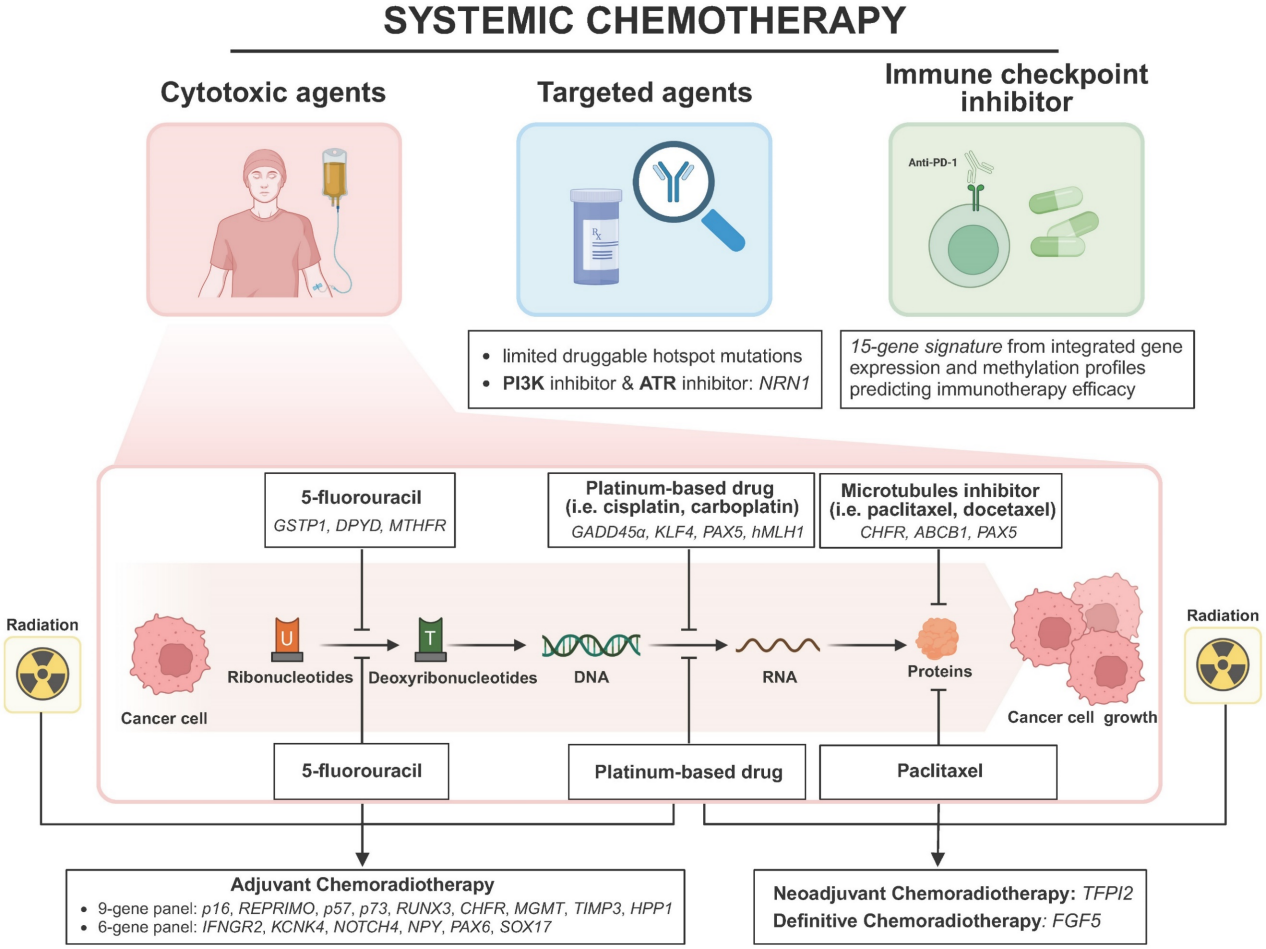
Overview of DNA methylation markers in ESCC systemic chemotherapy, including cytotoxic agents, targeted agents and immune checkpoint inhibitors. Abbreviations: PI3K, phospho-inositol-3 kinase; ATR, ataxia telangiectasia and Rad3-related protein kinase. Created in BioRender. Li, YK. (2025) https://BioRender.com/d45q992 (accessed on 25 January 2025).

As mentioned in the above review, DNA methylation has been implicated in silencing apoptotic genes, disrupting DNA repair mechanisms, dysregulating cell cycle and even enhancing drug efflux, all of which contribute to chemotherapy resistance. Notably, these specific genes epigenetically modified by DNA hypermethylation regulate cancer response to systemic chemotherapy, making them potential predictive markers for therapy response, as shown in **Figure 2**.

In 2006, Hamilton *et al.* firstly identified a combined 9-gene panel (*p16, REPRIMO, p57, p73, RUNX-3, CHFR, MGMT, TIMP-3,* and *HPP1*) to predict adjuvant chemotherapy (DDP and 5-FU) responses based on methylation levels from 12 ESCC patients and 23 EAC patients 116. Among these genes, *REPRIMO* and *p16*, key regulators of the cell cycle, exhibited significantly higher methylation rates in esophageal cancer patients non-responsive to chemoradiation, and promoter methylation of both genes impairs the ability to arrest at the G2/M checkpoint, where cisplatin exerts their effects, leading to chemoresistance 116, 117. The study conducted by Chang *et al.* in 2017 stands out for establishing a six-CpG panel, including *IFNGR2*, *KCNK4*, *NOTCH4*, *NPY*, *PAX6*, and *SOX17*, which predicts treatment response to chemoradiation (DDP, 5-FU and concurrent radiotherapy). This panel's risk score equation, derived from the methylation status of selected genes in 91 ESCC patients, demonstrated a remarkable power in discriminating poor responders from good responders, with an AUC of 0.930, underscoring its potential in personalized medicine 118. Iwabu *et al.* identified *FGF5* as an oncogenic growth factor and its methylation as a predictive marker for definitive chemoradiotherapy (dCRT) sensitivity from 117 ESCC patients, with low methylation linked to poor response (sensitivity 45%, specificity 90%), highlighting its potential clinical utility. FGF5 could be induced by dCRT in ESCC when it is unmethylated, supporting cell survival and leading to resistance 119. In a comprehensive study by Salta *et al.* to identify DNA methylation-based biomarkers for early detection and prediction of response to therapy in ESCC patients, the methylation of *GPX3* has been identified as a potential marker for predicting responses to therapy. *GPX3* encodes glutathione peroxidase, which mitigates oxidative damage. Methylation-induced inactivation, along with compensatory overexpression of other antioxidants and increased genomic instability, further enhances cisplatin resistance in cancer cells 120. Based on molecular profiles from 36 ESCC patients in 2021, de Klerk *et al.* revealed that *TP63* amplification and *TFPI2* gene promoter methylation are associated with unfavorable responses to neoadjuvant chemoradiotherapy which consists of carboplatin, paclitaxel and concurrent radiotherapy 121. Huang *et al.* demonstrated that *PON3* hypermethylation downregulates its expression, contributing to multidrug resistance in ESCC, although the underlying mechanisms remain to be further elucidated 122. The study by Min *et al.* sheds light on the epigenetic evolution of acquired resistance to combination therapy (cisplatin and verapamil) in ESCC. *SLC8A3* was identified as a potential multidrug resistance gene from 6 ESCC patients' WGBS results, whose promoter descending methylation dynamics were associated with resistance to chemotherapy 123.

Other findings about methylation markers in predicting platinum-based drug efficacy are as follows. Wang *et al.* linked *GADD45α* overexpression to promoter hypomethylation, affecting cisplatin sensitivity through the disruption of apoptotic pathways 108. Lin *et al.* demonstrated that long-term cisplatin exposure induces *OCT1* methylation, causing cisplatin resistance 124. Both *KLF4* and *PAX5* gene methylation were discovered to predict cisplatin sensitivity and clinical outcomes. KLF enhances cisplatin sensitivity in ESCC cells through apoptosis induction and cell cycle arrest, whereas PAX5 contributes to cisplatin resistance by regulating GLUT1, a known chemoresistance factor in cancer cells 103, 125. Cao *et al.* confirmed the role of *hMLH1* promoter methylation in cisplatin resistance through mismatch repair deficiency, suggesting demethylation treatment may enhance conventional chemotherapy efficacy 126.

About the response to 5-fluorouracil, *GSTP1* encoding a detoxifying enzyme was found to be methylated by DNMTs which were recruited by the long noncoding RNAs LINC01270 and LINC01419, thereby reducing the chemosensitivity of ESCC to 5-FU 127, 128. Meanwhile, dihydropyrimidine dehydrogenase (DPYD) as a 5-FU degrading enzyme, was repressed by LINC00261 overexpression via the methylation-dependent manner, ultimately enhancing the 5-FU response in ESCC 129. Moreover, *MTHFR* methylation mediated by lncRNA HOTAIR inhibits 5-FU sensitivity by regulating the systemic exposure of ESCC cells to 5-FU, suggesting that targeting HOTAIR or *MTHFR* could potentially overcome chemoresistance 130.

As for the efficacy of microtubule inhibitors, Yun *et al.* reported that *CHFR* methylation sensitizes ESCC cells to docetaxel and paclitaxel. This occurs because the CHFR protein's role at the prophase checkpoint, where it delays mitotic entry in response to microtubule poisons, thereby attenuating the cytotoxic efficacy of microtubule inhibitors 46. Sumarpo *et al.* found that *ABCB1* gene amplification and promoter demethylation contribute to the acquisition of taxane resistance in ESCC cell lines 131. Additionally, Zhang *et al.* revealed *PAX5* as a tumor suppressor gene regulated by promoter methylation, sensitizing ESCC cell lines to docetaxel and 5-FU by promoting p53 signaling activity 132.

With regards to molecular-targeted therapies, few studies on ESCC exist, most of which target EGFR and limited druggable hotspot mutations. Du *et al.* found that NRN1 methylation is a sensitive marker for PI3K-Akt-mTOR and ATR inhibitors, implying that demethylation of NRN1 could enhance the efficacy of these inhibitors in ESCC 133.

Recent multiple randomized studies have shown that immune checkpoint inhibitors (ICIs), especially PD-1/PD-L1 inhibitors, exhibit antitumor activity in advanced ESCC 134, 135. Nonetheless, the predictive value of existing maker for immunotherapy like PD-L1 status in ESCC is unsatisfactory 136. Identifying sensitive and solid predictive markers is essential to improve response and prognosis. Zheng *et al.* integrated gene expression and DNA methylation profiles to characterize two molecular subtypes of ESCC associated with distinct immune-related pathways and clinical outcomes. The 15-gene expression signature could predict the response rate to immunotherapy 137.

Researches on methylation biomarkers for predicting therapeutic response in ESCC are still ongoing. The studies mentioned above contribute to stratifying ESCC patients for personalized therapy and provide new strategies for overcoming chemotherapeutic drug resistance.

### DNA methylation as a therapeutic target in ESCC

DNA methylations differ from genetic alterations by being reversible, making them promising targets for therapeutic intervention 138. DNA methyltransferases (DNMTs), including DNMT1, DNMT3A, and DNMT3B, catalyze DNA methylation process. Nucleoside analogs such as 5-azacytidine and its derivative 5-aza-2'-deoxycytidine (decitabine) inhibit DNMT activity and are currently approved for treating myelodysplastic syndrome (MDS), significantly improving patient survival. Theoretically, DNMT inhibitors can reverse the hypermethylation-mediated silencing of tumor suppressor or other functional genes. Consequently, multiple preclinical and clinical trials are carried out to assess the efficacy of these agents in solid tumors, including ESCC 139, 140. To be noted, in a phase Ib/II study by Chen et al. low-dose decitabine-primed chemoimmunotherapy re-sensitized resistant cancer cells and showing promising safety and efficacy in ESCC patients, suggesting DNMT inhibitor as a potential therapeutic agent in relapsed/refractory ESCC patients 140. Two independent researches both highlighted that low-dose decitabine epigenetically upregulates MAGE-A antigens, enhancing T cell-mediated tumor recognition and antigen-specific responses. These findings suggest that combining decitabine with immunotherapy may be a promising strategy for treating advanced ESCC 141, 142.

Recently Chang et al. demonstrated that Nutlin-3, a murine double min 2 (MDM2) small molecule inhibitor, could function as a novel DNMT inhibitor via enhancing p53 and RB expression, making it a promising DNMT inhibitor for sensitizing chemoradiation-resistant ESCC to therapy 143. Besides that, natural compounds have demonstrated potential as DNMT inhibitors. Fang et al. reported that (-)-epigallocatechin-3-gallate (EGCG), a major green tea extract, inhibits DNMT1 activity, reversing methylation and restoring the expression of tumor suppressor genes such as *CDKN2A*, *RARB*, *MGMT*, and *MLH1* in ESCC cell lines 144. Similarly, genistein, a soy-derived isoflavone, was found to reverse the hypermethylation of TSGs via a direct inhibition of DNA methyltransferase, suggesting these natural extracts could contribute to the chemoprevention of ESCC 145. Huang *et al.* indicated that black raspberries (BRBs) could reduce DNMT1 and DNMT3b mRNA levels, thereby demethylating Sfrp4, inhibiting WNT signaling and suppressing esophageal tumorigenesis in rats, a model akin to human ESCC. This suggests that BRBs may prevent the aberrant DNA methylation in ESCC development, offering a natural approach to target epigenetic alterations in cancer prevention 146.

Nevertheless, currently available DNMT inhibitors are not sufficiently selective, leading to off-target adverse effects including global DNA hypomethylation, oncogene activation and increased genomic instability. Therefore, further researches are needed to develop targeted therapies that specifically reverse the hypermethylation of tumor suppressor genes, thereby improving therapeutic efficacy.

In addition to DNMTs, TET1 as a CpG demethylase was reported to have the catalytic domain to inhibit the CpG methylation of TSG promoters and reactive their expression 147. Interestingly, *TET1* underwent promoter CpG methylation-mediated silencing in human cancers including ESCC, leading to elevated methylation levels in tumor cells via a DNA methylation feedback loop, which suggests targeting *TET1* as a therapeutic target is worth further research. UHRF1, a multidomain nuclear protein that recruits DNMT1 to maintain DNA methylation has also been identified as a potential therapeutic target in ESCC patients, particularly those with elevated *UHRF1* expression 148.

Regarding the repurposing of non-cancer drugs, there have been some interesting findings. In a pre-clinical trial, Liu et al. showed that the nonsteroidal anti-inflammatory drug celecoxib demethylated TSGs and boosted apoptosis, highlighting its potential as an epigenetic therapy 149. The study by Wang *et al.* revealed that metformin, a first-line drug for diabetes, could counteract nicotine-upregulated CHRNA7 expression in ESCC by enhancing promoter hypermethylation, thereby inhibiting JAK2/STAT3/SOX2 signaling pathway and tumor progression. The finding underscores the therapeutic potential of metformin in modulating epigenetic changes and offers a novel strategy for ESCC treatment, especially in smoking patients 150.

## Discussion and Perspectives

Despite continuous advancements and improvements in multidisciplinary treatments, esophageal squamous cell carcinoma remains one of the most lethal malignancies in China and worldwide. DNA methylation, a major component of epigenetic modifications, plays a crucial role in the initiation and progression of ESCC. Compared to message RNA and protein, DNA is more stable biochemically, and tumor-derived DNA can be readily obtained from patient specimens including plasma, stool, sputum and urine after being released into the circulation. Moreover, techniques to detect DNA methylation like pyrosequencing, methylated DNA immunoprecipitation sequencing (MeDIP-Seq) and methylation-specific PCR (MSP), provide significant technical advantages. Taken together, the detection of aberrantly methylated genes can be exploited as potent predictive methods for clinical translation.

The first part of this review provides a comprehensive summary of all aberrantly methylated genes identified in ESCC to date and their associated characteristics. Previous studies and reviews have repeatedly highlighted the clinical applications of DNA methylation biomarkers in ESCC, particularly for early screening, early diagnosis and prognostic prediction. However, given the limited efficacy of systemic chemotherapy in ESCC and lack of reliable predictive biomarkers and novel therapeutic targets, there is a pressing need for further exploration. From the perspectives of significance and necessity, the latter part of this review meticulously discusses the predictive value of aberrant DNA methylation for therapeutic efficacy in systemic treatments and its potential as a promising therapeutic target.

Through extensive review of the literature, we have found that the major obstacle preventing DNA methylation markers from becoming truly useful biomarkers in ESCC is the lack of suitable large-scale, prospective clinical trial validation. In the future, we will focus on more large-sample prospective cohort studies and even initiate large-scale investigator-driven clinical trials based on our promising preliminary findings to identify and verify DNA methylation markers that can genuinely improve treatment outcomes and prognosis in clinical settings. Besides that, we have observed that epigenetic patterns like DNA methylation, exhibit considerable plasticity and undergo dynamic changes during tumor progression or under therapeutic selection pressure. Therefore, it is essential to adopt a more dynamic perspective to evaluate DNA methylation markers, paying close attention to their temporal changes. By flexibly interpreting thresholds and incorporating these dynamic processes, we can achieve more precise treatment and timely therapeutic adjustments.

ESCC exhibits a relatively high proportion of methylation abnormalities, and epigenetic therapies targeting these aberrations hold substantial potential, with multiple clinical studies currently underway. It is important to note, however, that epigenetic therapies generally lack specificity, leading to considerable off-target effects and toxicity. As a result, most methylation-targeting drugs are currently administrated at low doses and are used in combination with conventional chemotherapy agents. Future research needs to develop more specific methylation-targeting drugs to enhance both efficacy and safety, as well as exploring the synergistic effects of these drugs with other conventional therapies, with the aim of significantly improving treatment outcomes and reversing drug resistance. It is anticipated that large-scale clinical trials in ESCC along with further exploration of the regulatory mechanisms of DNA methylation will discover more effective predictors of therapeutic response and identify novel therapeutic targets for eventual clinical application.

## Conclusion

This review underscores the pivotal role of DNA methylation in ESCC, particularly its involvement in systemic chemotherapy. The key findings highlight the identification of numerous aberrantly methylated genes that disrupt critical pathways in ESCC. Clinically, DNA methylation markers hold promise for predicting therapeutic responses, though their utility is hindered by insufficient validation and off-target effects. Future research should prioritize large-scale validation of methylation markers through prospective trials and the development of more targeted epigenetic therapies. Integrating dynamic changes in methylation during treatment could enhance precision medicine. Further exploration of methylation mechanisms may reveal new therapeutic targets and improve clinical outcomes in ESCC.

## Figures and Tables

**Table 1:** Hypermethylated functional genes in esophageal squamous cell carcinoma

Classification	Year	Gene Symbol	Description	Major functions	Location	Methylation rate in tumor (%)	References
DNA damage repair genes	1998	*FHIT*	Fragile Histidine Triad Diadenosine Triphosphatase	DNA damage response, cell-cycle regulation	3p14.2	14-33, 50	33, 34
	2003	*MGMT*	O-6-Methylguanine-DNA Methyltransferase	DNA damage repair	10q26	27-72	35, 36
	2005	*MLH1*	MutL Homolog 1	DNA mismatch repair, cell-cycle regulation	3p22.3	3-62	37-39
	2005	*MSH2*	MutS Homolog 2	DNA mismatch repair, cell-cycle regulation	2p21	29-32	40
	2006	*BRCA1*	BRCA1 DNA Repair Associated	DNA damage repair, cell-cycle regulation, transcriptional activator	17q21.31	26-28	38
	2015	*HIC1*	HIC ZBTB Transcriptional Repressor 1	DNA damage response, apoptosis, Wnt signaling pathway, tumor suppressor	17p13.3	84.2	41
	2018	*EYA4*	EYA Transcriptional Coactivator and Phosphatase 4	DNA damage repair, migration and invasion, epithelial-mesenchymal transition, AKT signaling pathway, tumor suppressor	6q23.2	78-85.7	42
Cell cycle control genes	1999	*CDKN2A* (p16^INK4a^)	Cyclin Dependent Kinase Inhibitor 2A	Cell-cycle regulation, tumor suppressor	9p21	12-88	35, 37, 43-45
	1999	*CDKN2B* (p15^INK4b^)	Cyclin Dependent Kinase Inhibitor 2B	Cell-cycle regulation	9p21	13-18	43
	2003	*RASSF1*	Ras Association Domain Family Member 1	Cell-cycle regulation, apoptosis	3p21.3	14-53	34
	2015	*CHFR*	Checkpoint With Forkhead and Ring Finger Domains	Cell cycle and apoptosis, tumor suppressor	12q24.33	45	46
Pro-apoptotic genes	2005	*UCHL1* *(PGP9.5)*	Ubiquitin C-Terminal Hydrolase L1	Ubiquitin regulation, cell growth inhibition, apoptosis, tumor suppressor	4p13	42	47
	2006	*DAPK1*	Death Associated Protein Kinase 1	Cell survival, apoptosis, and autophagy	9q21.33	26-38	38, 44
	2010	*ZNF382*	Zinc Finger Protein 382	Pro-apoptotic transcription factor, tumor suppressor	19q13.12	89	48
	2011	*RUNX3*	RUNX Family Transcription Factor 3	Cellular growth and differentiation, Notch and TGF-β signaling pathway, tumor suppressor	1p36.11	35.8-51.4	49, 50
Metastasis suppressor genes	2003	*CADM1* *(TSLC1)*	Cell Adhesion Molecule 1	Migration and invasion, cell proliferation, tumor suppressor	11q23.3	50	51
	2004	*CDH1* *(E-cadherin)*	Cadherin 1	Cell adhesion, proliferation, metastasis, epithelial-mesenchymal transition	16q22.1	14-61	38, 44, 52
	2004	*CDH13*	Cadherin 13	Cell adhesion and recognition, tumor growth, cell cycle, tumor suppressor	16q23.3	14-39.4	53
	2006	*PCDH10*	Protocadherin 10	Cell-cell interaction, cell migration, tumor suppressor	4q28.3	51-81	54
	2006	*CLDN3*	Claudin 3	Cell-cell adhesion, cell tight junctions	7q11.23	69	35
	2008	*DCC*	DCC Netrin 1 Receptor	Cell adhesion, cell proliferation and differentiation, apoptosis, tumor suppressor	18q21.2	74	55
	2010	*UPK1A*	Uroplakin 1A	Cell motility, metastasis, tetraspanin superfamily, tumor suppressor	19q13.12	62	56
	2011	*CLDN4*	Claudin 4	Cell-cell adhesion, cell tight junctions	7q11.23	41.7	57
	2012	*CDH11*	Cadherin 11	Cell adhesion, migration and invasion, Wnt/β-catenin signaling pathway	16q21	93	58
Growth factor response-related genes	2003	*RARB*	Retinoic Acid Receptor Beta	Growth factor response, cellular growth and differentiation	3p24	25, 39, 67-70	34, 35, 44
	2005	*RBP1*	Retinol Binding Protein 1	Retinoid signaling	3q23	17.9-31	35, 59
	2005	*RARRES1* *(TIG1)*	Retinoic Acid Receptor Responder 1	Retinoid signaling	3q25.32	17.9	59
	2007	*CRABP1*	Cellular Retinoic Acid Binding Protein 1	Cell cycle, proliferation, tumor suppressor	15q25.1	52.8	60
	2011	*SOCS1*	Suppressor Of Cytokine Signaling 1	Negative regulator of JAK/STAT pathway and interferon signaling, tumor suppressor	16p13.13	45.3	61
Wnt signaling related genes	2007	*APC*	APC Regulator of WNT Signaling Pathway	Wnt signaling pathway, cell polarity and chromosome segregation	5q21-q22	27-46	37
	2007	*SFRP1*	Secreted Frizzled Related Protein 1	Wnt signaling modulator	8p11.21	29.6-64.3	37, 44, 50
	2007	*SFRP2*	Secreted Frizzled Related Protein 2	Wnt signaling modulator	4q31.3	19.6	37
	2007	*WIF1*	WNT Inhibitory Factor 1	Wnt signaling pathway inhibitor	12q14.3	27-35	44, 62
	2011	*DKK3*	Dickkopf WNT Signaling Pathway Inhibitor 3	Wnt signaling inhibitor, tumor suppressor	11p15.3	37.4	50
	2012	*SOX17*	SRY-Box Transcription Factor 17	Cell proliferation, Wnt signaling pathway, transcription regulator	8q11.23	65	63
Genes with tumor suppressive functions	2003	*ECRG4*	ECRG4 Augurin Precursor	Cell proliferation, cell cycle, senescence, tumor suppressor	2q12.2	80	64
	2003	*PRSS3* *(Trypsinogen 4)*	Serine Protease 3	Proteolytic activity, tumor suppressor	9p13.3	50	65
	2003	*VHL*	Von Hippel-Lindau Tumor Suppressor	Ubiquitin ligase component, tumor suppressor	3p25.3	36	34
	2005	*MT3*	Metallothionein 3	Cellular growth inhibition, intracellular metal homeostasis	16q13	52	66
	2006	*GRIN2B* *(NMDAR2B)*	Glutamate Ionotropic Receptor NMDA Type Subunit 2B	Apoptosis, signal transduction, tumor suppressor	12p13.1	95	67
	2007	*DLC1*	DLC1 Rho GTPase Activating Protein	Cytoskeleton organization, Ras-mediated signaling pathways, cell adhesion, cell cycle, tumor suppressor	8p22	51	68
	2007	*CDX2*	Caudal Type Homeobox 2	Cellular growth and differentiation, transcription factor	13q12.2	49	69
	2007	*PLCD1*	Phospholipase C Delta 1	Phospholipase, cell proliferation, cell cycle, migration and invasion, tumor suppressor	3p22.2	38	70
	2007	*ADAMTS18*	ADAM Metallopeptidase with Thrombospondin Type 1 Motif 18	Tumor microenvironment modulator, cell proliferation, tumor suppressor	16q23	52-88	71
	2007	*EP300*	E1A Binding Protein P300	Transcription regulator activity, tumor invasion and metastasis	22q13.2	42	72
	2008	*GNG7*	G Protein Subunit Gamma 7	Signal transduction, tumor suppressor	19p13.3	33.3	73
	2008	*SST*	Somatostatin	Somatostatin hormone, tumor suppressor	3q27.3	53.8	74
	2008	*HOPX*	HOP Homeobox	Tumor suppressor, serum response factor-related pathway	4q12	50	75
	2008	*ITGA4*	Integrin Subunit Alpha 4	Cell attachment to ECM, signal transduction, tumor suppressor	2q31.3	21	44
	2008	*IRF8*	Interferon Regulatory Factor 8	Transcriptional factor, IFN-γ signaling pathway, immune response, cellular growth and differentiation, tumor suppressor	16q24.1	58	76
	2008	*ENG*	Endoglin	TGF-β signaling pathway, angiogenesis, tumor suppressor	9q34.11	46.2	77
	2009	*KAT2B* *(PCAF)*	Lysine Acetyltransferase 2B	Transcription regulator activity, histone acetylation, cell cycle, tumor suppressor	3p24.3	70	78
	2010	*NEFH*	Neurofilament Heavy Chain	Mitochondrial function, glycolysis, Akt/β-catenin pathway, tumor suppressor	22q12.2	65	79
	2011	*HSPB2*	Heat Shock Protein Family B (Small) Member 2	Stress response and resistance, heat shock protein activity, tumor suppressor	11q23.1	95.7	80
	2012	*PTK6*	Protein Tyrosine Kinase 6	Cell proliferation, cellular differentiation, apoptosis, migration and invasion, tumor suppressor	20q13.33	40	81
	2012	*TFPI2*	Tissue Factor Pathway Inhibitor 2	Proteolytic activity, ECM degradation, tumor suppressor	7q21.3	67	82
	2012	*RAB25*	RAB25, Member RAS Oncogene Family	Cell survival, migration, invasion and angiogenesis, MAPK/ERK signaling pathway, tumor suppressor	1q22	75-100	83
	2013	*DIRAS1*	DIRAS Family GTPase 1	Apoptosis, cell motility, ERK and p38 MAPK signaling pathways, tumor suppressor	19p13.3	40	84
	2014	*DACH1*	Dachshund Family Transcription Factor 1	TGF-β signaling, transcription factor, tumor suppressor	13q21.33	61.5	85
	2014	*RASSF10*	Ras Association Domain Family Member 10	Cell proliferation, cell cycle regulation, microtubule stability, tumor suppressor	11p15.3	44.3	86
	2014	*ADAMTS8*	ADAM Metallopeptidase With Thrombospondin Type 1 Motif 8	Cell proliferation, apoptosis, EGFR-MEK-ERK signaling pathway, tumor suppressor	11q24.3	22	87
	2014	*SPINT2*	Serine Peptidase Inhibitor, Kunitz Type 2	Cell proliferation, apoptosis, tumor suppressor	19q13.2	52.08	88
	2015	*RASSF5A*	Ras Association Domain Family Member 5A	Apoptosis, tumor suppressor	1q32.1	47.1	89
	2017	*BIN1*	Bridging Integrator 1	Cell proliferation, tumor suppressor	2q14.3	62	90
	2017	*PAX1*	Paired Box 1	Transcription factor, tumor suppressor	20p11.22	96-100	91, 92
	2018	*RHCG*	Rh Family C Glycoprotein	Ammonium transporter, invasion, metastasis, NF-κB signaling pathway, tumor suppressor	15q26.1	86.4	93
	2019	*CHL1*	Cell Adhesion Molecule L1 Like	Cell proliferation, cell cycle, metastasis, AKT signaling pathway, tumor suppressor	3p26.3	59.0	94
	2019	*SEMA3B*	Semaphorin 3B	Cell proliferation, invasion, tumor suppressor	3p21.31	54.3	95
	2024	*PREX2*	Phosphatidylinositol-3,4,5-Trisphosphate Dependent Rac Exchange Factor 2	Migration, invasion, tumor suppressor	8q13.2	14.6	96
Other functional genes	2006	*GATA4*	GATA Binding Protein 4	Zinc-finger transcription factor	8p23.1	61	97
	2006	*GATA5*	GATA Binding Protein 5	Zinc-finger transcription factor	20q13.33	32	97
	2006	*MT1G*	Metallothionein 1G	Cellular stress response, metal metabolism, detoxification	16q13	62	35
	2007	*TAC1*	Tachykinin Precursor 1	Neurotransmitter	7q21.3	50	98
	2007	*CACNA1G*	Calcium Voltage-Gated Channel Subunit Alpha1 G	Calcium-dependent process, hormone or neurotransmitter release, cell proliferation, cell motility and cell death	17q21.33	23.2	37
	2008	*SCGB3A1* *(HIN-1)*	Secretoglobin Family 3A Member 1	Signal transduction, cytokine activity, cellular growth	5q35.3	50-72	99
	2011	*HLA-I*	Major Histocompatibility Complex, Class I	Immune response, immune evasion, metastasis	6p21-22	70.1	100
	2011	*PTX3*	Pentraxin 3	Innate immune defense, inflammatory reactions, angiogenesis	3q25.32	85	101
	2011	*GPX3*	Glutathione Peroxidase 3	Antioxidative defense	5q33.1	54.8-71.4	102
	2017	*PAX5*	Paired Box 5	Cell proliferation, cell cycle, chemosensitivity, transcription factor	9p13.2	85.9	103
	2017	*ZNF582*	Zinc Finger Protein 582	Transcriptional regulator	19q13.43	85.7-93.2	91, 92
	2018	*ZNF132*	Zinc Finger Protein 132	Cell proliferation, apoptosis, migration, transcription factor	19q13.43	70.8	104
	2018	*SLC22A3*	Solute Carrier Family 22 Member 3	Antioxidative defense, DNA damage repair	6q25.3	NA	105
	2019	*SOX1*	SRY-Box Transcription Factor 1	Transcription factor	13q34	89.2	92

## Abbreviations

ESCC: Esophageal squamous cell carcinoma
EAC: Esophageal adenocarcinoma
DDP: Cisplatin
5-FU: 5-fluorouracil
FDA: Food and drug administration
TSG: Tumor suppressor gene
LINEs: Long interspersed nuclear elements
SINEs: Short interspersed nuclear elements
IL6: Interleukin 6
dCRT: Definitive chemoradiotherapy
WGBS: Whole genome bisulfite sequencing
DNMTs: DNA methyltransferases
DPYD: dihydropyrimidine dehydrogenase
ATR: Ataxia Telangiectasia and Rad3-related protein kinase
ICI: Immune checkpoint inhibitor
MeDIP-Seq: Methylated DNA immunoprecipitation sequencing
MSP: Methylation-specific polymerase chain reaction
